# Why Is Apolipoprotein CIII Emerging as a Novel Therapeutic Target to Reduce the Burden of Cardiovascular Disease?

**DOI:** 10.1007/s11883-016-0614-1

**Published:** 2016-09-09

**Authors:** Marja-Riitta Taskinen, Jan Borén

**Affiliations:** 1 Heart and Lung Centre, Helsinki University Central Hospital and Research Programs’ Unit, Diabetes & Obesity, University of Helsinki, Helsinki, Finland; 2 Department of Molecular and Clinical Medicine, University of Gothenburg and Sahlgrenska University Hospital, Gothenburg, Sweden; 3 Wallenberg Laboratory, Sahlgrenska University Hospital, Gothenburg, Sweden

**Keywords:** apoC-III, Lipoproteins, Triglycerides, Remnants, CVD

## Abstract

ApoC-III was discovered almost 50 years ago, but for many years, it did not attract much attention. However, as epidemiological and Mendelian randomization studies have associated apoC-III with low levels of triglycerides and decreased incidence of cardiovascular disease (CVD), it has emerged as a novel and potentially powerful therapeutic approach to managing dyslipidemia and CVD risk. The atherogenicity of apoC-III has been attributed to both direct lipoprotein lipase-mediated mechanisms and indirect mechanisms, such as promoting secretion of triglyceride-rich lipoproteins (TRLs), provoking proinflammatory responses in vascular cells and impairing LPL-independent hepatic clearance of TRL remnants. Encouraging results from clinical trials using antisense oligonucleotide, which selectively inhibits apoC-III, indicate that modulating apoC-III may be a potent therapeutic approach to managing dyslipidemia and cardiovascular disease risk.

## Introduction

Apolipoprotein C-III (apoC-III) was first identified by Brown et al. in 1969 as a regulator of triglyceride-rich lipoproteins (TRLs) in the circulation [1]. In the blood, it is mainly present in TRLs (chylomicrons and VLDL) and, to a lesser extent, in LDL and HDL particles. Shortly after its discovery, it was identified as an inhibitor of lipoprotein lipase (LPL) activity and a key regulator of TRL concentrations in plasma [2, 3]. In addition to its potent inhibitory capacity on LPL-mediated lipolysis, apoC-III has also been shown to facilitate hepatic VLDL assembly and secretion. ApoC-III is not only a critical modulator of TRLs but also contributes to the atherogenicity of low- and high-density lipoprotein (LDL and HDL) particles [4–7]. Substantial evidence has emerged to indicate that apoC-III has several additional functions, including modulation of endothelial function and inflammation [8, 9].

The connection of apoC-III with hypertriglyceridemia and CVD risk has been confirmed in extensive animal and humans studies [10, 11]. Furthermore, given that TRL remnants are now recognized as a causal risk factor for CVD and that loss-of-function mutations of apoC-III are characterized by a robust lowering of serum triglycerides and reduced CVD risk [12, 13•, 14••, 15], apoC-III has been identified as an attractive target for the prevention of CVD. This review evaluates the role of apoC-III in the pathogenesis of CVD and summarizes its role as a novel therapeutic target.

### Regulation of Apoc-III

ApoC-III is the most abundant C-apolipoprotein in humans. It is found not only in triglyceride-rich chylomicrons and very low-density lipoproteins (VLDLs) but also in HDL. In humans, apoC-III is mainly expressed in hepatocytes and enterocytes. Its expression is chiefly regulated at the transcriptional level, and the regulatory sequences of the *APOC3* gene have been extensively described (reviewed in [16]). The *APOC3* gene contains two regulatory elements that act as a common enhancer for a gene cluster on 11q23 that regulates the expression of three apolipoprotein genes: *APOC3*, *APOA1*, and *APOA4* [16, 17].

Promoter analysis of the *APOC3* gene has identified binding sites for several transcription factors including the carbohydrate response element binding protein (ChREBP) [18–20]. Thus, glucose and plasma triglyceride metabolism are linked via the regulation of *APOC3* expression. Additional transcription factors that bind to the *APOC3* gene include the nuclear receptors hepatocyte nuclear factor-4α (HNF-4α) [21] and liver X receptors (LXRs) [22]. Interestingly, the expression of *APOC3* is upregulated by glucose and downregulated by insulin. This contrasts with most other genes in the glycolytic and lipogenic pathways, which are regulated positively both by glucose and insulin [23]. It has, therefore, been proposed that the glucose-mediated expression of *APOC3* may contribute to lower lipolysis and consequently to an increase in peripheral glucose handling [20]. Substantial evidence indicates that dysregulation of the insulin signaling pathway via FoxO1 aggravates *APOC3* expression as the inhibitory effect of insulin on *APOC3* expression that is lost [24]. This mechanism may explain why apoC-III concentrations are increased in insulin-resistant states such as obesity, the metabolic syndrome, type 2 diabetes, and hypertriglyceridemia [10, 11, 25].

ApoC-III is a small protein (79 amino acid residues) that contains two amphipathic helices [26]. We still lack a detailed structure-function understanding of apoC-III, and it is not clear which regions are most important for attachment to lipids [26–29]. ApoC-III can undergo posttranslational modification on threonine 74, and three different glycoforms have been described with zero, one, or two sialic acids (termed apoC-III_0_, apoC-III_1_, and apoC-III_2_) [30]. The degree of sialylation of apoC-III has been reported to influence its function as apoC-III_2_ inhibits LPL-mediated hydrolysis of TRLs less efficiently than apoC-III_1_ [31], despite having an apparent twofold greater affinity for TRL than the other two apoC-III isoforms [32].

### Function of Apoc-III

In addition to inhibiting LPL-mediated lipolysis of TRLs, apoC-III binds surface heparan sulfate proteoglycans (HSPGs) on the liver and cellular receptors, including the HDL receptor SR-BI [33]. However, the significance of binding to SR-BI is still unclear.

#### Inhibition of LPL-mediated Lipolysis

Lipolysis of TRLs is mediated by LPL, which resides on cellular surfaces. ApoC-III has been shown in vitro to inhibit lipolysis by several mechanisms, including displacing lipoproteins from the negatively charged cell surface [34], inducing conformational changes in apoE and inhibiting the activation of LPL by displacing the LPL activator apoC-II from the lipoprotein surface [35]. In addition, studies in vivo with apoC-III transgenic mice have demonstrated that overexpression of apoC-III induces marked hypertriglyceridemia due to accumulation of remnant particles with increased apoC-III and decreased apoE content compared with controls [36].

#### Inhibition of Lipoprotein Binding to HSPGs and the LDL Receptor Family

ApoC-III displaces apoE from lipoprotein particles [37]. This displacement can either be complete or involve only the N-terminal domain of apoE [37]. Since apoC-III impairs the clearance of TRLs and their remnants, whereas apoE mediates their clearance (by binding to hepatic HSPG and the LDL receptor), the relative proportions of apoC-III and apoE on apoB-containing lipoproteins regulate the metabolism of these lipoproteins, which is reflected in LDL and HDL subspecies. This is a very complex area of research, and Dr Sacks has made significant contributions in clarifying the crucial roles of apoE and apoC-III in apoB-lipoprotein metabolism [6].

#### Human Kinetic Studies

Recent evidence from kinetic studies showed that dual metabolic defects contribute to elevated TRLs in the atherogenic dyslipidemia of abdominally obese men [38•, 39, 40]. In fact, reduced catabolism of TRLs resulting from increased levels of plasma apoC-III was shown to be a more important predictor of plasma triglycerides than increased secretion rate of TRLs driven by liver fat content [38•]. Indeed, kinetic studies in humans have also provided direct evidence to show that apoB-containing lipoproteins are removed significantly more slowly from the circulation if they contain apoC-III [41]. These results may indicate that apoC-III concentration may be a good biomarker of the residual risk of CVD in subjects with atherogenic dyslipidemia; this issue remains a major clinical problem in statin-treated subjects.

The finding that removal of TRLs from the circulation is a more important determinant of plasma TG than secretion of TRL is interesting, but it remains to be clarified whether the apoC-III-induced impaired clearance of VLDL particles in subjects with atherogenic dyslipidemia is predominantly due to reduced lipolytic capacity or to the impaired removal of TRL remnants by the liver. This question is of importance when considering the potential of apoC-III inhibition as a novel treatment modality. Because the lipid oxidation capacity of the liver is limited, a potential concern is that increased lipid load from the uptake of remnant particles may lead to excess fat accumulation in hepatocytes, resulting in non-alcoholic fatty liver disease [42].

Recently, apoC-III has also been implicated in VLDL secretion [43–45]. Overexpression of apoC-III in mice and in vitro resulted in increased secretion of triglyceride-rich VLDL particles [43]. Structure-function analysis of apoC-III has identified two domains in apoC-III that seem to influence the formation and secretion of VLDL [43]. However, inhibition of apoC-III synthesis by apoC-III antisense oligonucleotides (ASO) did not reduce VLDL secretion in mice [46] so the significance of apoC-III for VLDL secretion requires further study, particularly in humans.

### Measurements of ApoC-III Concentrations

Standardized clinical immunoassays for the measurements of apoC-III concentrations have not been easily available. The most common system to measure plasma apoC-III concertation has been the immunoturbidimetric assay provided by automatic autoanalyzers [47]. More recently, a sensitive sandwich ELISA to quantify apoC-III concentrations was developed for commercial use [48]. However, because apoC-III circulates in multiple glycoforms that are differentially distributed in lipoprotein fractions (VLDL, LDL, and HDL), the measurement of plasma apoC-III concentrations is not enough to understand the complexity of apoC-III in lipoprotein metabolism, particularly in the context of novel apoC-III ASO therapies. Therefore, it is critical that novel assays with high sensitivity and specificity be developed. Furthermore, to better understand the clinical importance of the different glycoforms, assays that can be easily performed in routine clinical chemistry laboratories are needed [5, 30, 49–51].

### Role of ApoC-III in Atherogenesis and Inflammation

**Fig. 1:**
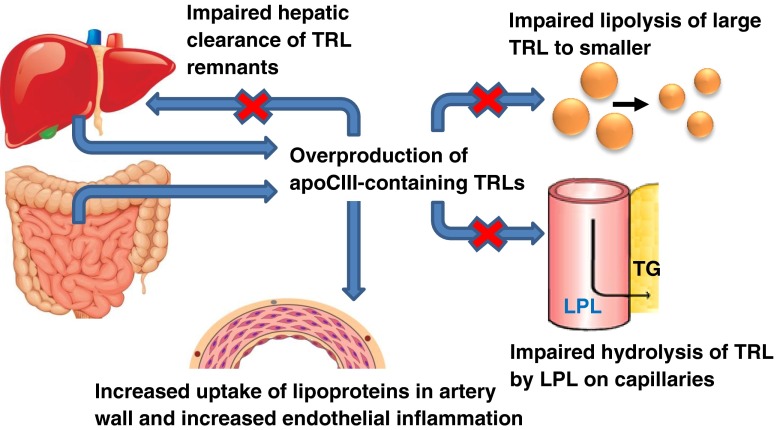
Proatherogenic action of apoC-III on lipid metabolism and atherogenicity. ApoC-III exerts strong atherogenic functions through both indirect and direct mechanisms. These include inducing oversecretion of apoC-III-containing triglyceride-rich lipoproteins (TRLs). These lipoproteins accumulate in the circulation by three mechanisms: (1) impaired lipolysis of large TRLs to smaller remnant particles, (2) impaired LPL-mediated lipolysis of TRLs on capillaries, and (3) impaired LPL-independent and LPL-dependent hepatic clearance of TRL remnants. In addition, apoC-III promotes proinflammatory responses in endothelial cells and monocytes and increases the binding affinity of LDL, leading to increased accumulation of atherogenic lipoproteins in the artery wall

Recent epidemiological studies have clearly demonstrated that apoC-III is associated with higher risk of CVD, but what makes apoC-III atherogenic? As discussed above, apoC-III regulates lipid metabolism through multiple mechanisms with adverse effects especially on TRLs and their remnants (Fig. 1). In addition to effects on lipid metabolism, apoC-III has been shown to influence atherogenesis by increasing the affinity of LDL for artery wall proteoglycans, and thus increasing accumulation of atherogenic lipoproteins in the vessel wall [7, 52–56]. The mechanism was elusive since apoC-III by itself does not bind artery wall proteoglycans. However, Hiukka et al. identified a potential explanation by demonstrating that apoC-III influences the lipid composition not only of TRLs but also of LDL [7]. They investigated subjects with type 2 diabetes and showed that LDL with a high apoC-III/apoB molar ratio is associated with a reduction in unesterified cholesterol, sphingomyelin, and ceramide, but not phosphatidylcholine [7]. These changes in lipid composition associate with higher membrane fluidity, thus allowing apoB to acquire a conformation that is more favorable for proteoglycan binding [7, 57].

Following subendothelial retention in the artery wall, LDL is exposed to several enzymes, including sphingomyelinases (SMase), that promote aggregation and fusion of retained lipoproteins [58]. Interestingly, apoC-III is a SMase activator and apoC-III-enriched LDL displays increased susceptibility to hydrolysis and aggregation by SMase [7, 59]. ApoC-III has also been linked to inflammation as apoC-III alone, or as a component of TRLs and LDL, induces activation of adhesion molecules and the proinflammatory nuclear factor-kB in monocytes and endothelial cells [60, 61].

### ApoC-III and CVD Risk

Data from several observational studies show that plasma apoC-III levels associate with manifestations of CVD and the progression of coronary artery disease [5, 11]. Epidemiological studies have further demonstrated that apoC-III in plasma, VLDL, and LDL predicts CVD [10, 11, 62–65]. Importantly, in a 15-year follow-up study, age- and sex-adjusted apoC-III concentrations were shown to predict CVD mortality [65]. Recently, Qamar et al. reported that apoC-III levels in patients with type 2 diabetes patients (*n* = 1422) associate with elevation of triglycerides and higher coronary artery calcification (CAC) score, a measure of subclinical atherosclerosis [66•]. Notably, this relationship between apoC-III and CAC was found to be triglyceride dependent. The positive association of apoC-III levels with CVD has recently been confirmed in a meta-analysis of published data [5]. The finding of an inverse relationship between plasma apoC-III levels and incident CVD reported in a large case–control study of type 2 diabetic subjects (*n* = 1123) was unexpected [67]. Normally, plasma apoC-III correlates strongly with triglyceride levels. However, in this cohort of type 2 diabetics, this correlation was, at best, only modest [67]. Overall, the association of apoC-III with CVD risk has been reported to be independent of statin use, suggesting that apoC-III could become a marker for the residual risk of CVD in statin-treated patients with atherogenic dyslipidemia [63, 66•, 68].

In addition, higher apoC-III content in both VLDL and LDL shows consistent links with CVD events, highlighting the multifaceted role of apoC-III as a proatherogenic apolipoprotein (7). The available data on apoC-III in HDL particles are heterogeneous and less clear. However, two studies reported that apoC-III in HDL particles is associated with increased CHD risk [63, 69]. Remaining important questions are how apoC-III modifies HDL to become proatherogenic and if this may explain the unexpected findings in subjects with type 2 diabetes reported by Looker et al. [67].

The rapid development of molecular genetic technology has provided the tools to determine whether apoC-III has a causal role as a proatherogenic protein. Recently, a comprehensive meta-analysis of three main apoC-III polymorphisms (SstI, T-455C, and C-482T) known to associate with hypertriglyceridemia in mice [36] reported that two polymorphisms (SstI and T-455C) significantly increased the susceptibility to CHD in humans [70]. The association of the apoC-III T-455C with increased CVD risk was confirmed in another meta-analysis [71]. In contrast, some evidence emerged that rare variants of apoC-III associated with low triglycerides and high HDL cholesterol [72, 73]. In line with the lipid profile of low triglycerides and high HDL cholesterol, a lifelong deficiency of apoC-III has been shown to associate with reduced subclinical atherosclerosis, being cardioprotective [74]. Together, these data support strongly the link of polymorphisms in apoC-III with serum levels of triglycerides, as well as with either promotion or prevention of CVD.

Two large Mendelian randomization studies have recently shown that polymorphisms in the *APOC3* gene were associated with decreased incidence of CVD [13•, 14••]. In these cohorts, three loss-of-function mutations in the *APOC3* gene were identified and the heterozygote carriers of these rare variants had about 39–44 % lower serum triglyceride levels, about 46 % lower apoC-III levels and a 36–40 % lower risk of CHD than non-carriers. Furthermore, the reduced risk of CVD was independent of statin use. Recently, carriers of the three loss-of-function mutations of *APOC3* were shown to exhibit lower triglyceride levels together with higher HDL cholesterol and a decreased burden of coronary arterial calcification (decreased median CAC score), but no difference was observed in either carotid plaque or carotid intima-media thickness [68].

### Potential Therapies to Reduce ApoC-III Plasma Levels

Currently available tools to reduce apoC-III levels and consequently TRLs and their remnants remain limited. Peroxisome proliferator activated receptor (PPAR) alfa agonists are known to downregulate the expression of apoC-III and to reduce its plasma levels in both animal and human studies [75]. Given the fact that apoC-III levels are increased in people with type 2 diabetes [76] and that fibrates reduce apoC-III levels [75, 77], it is surprising that so far no data exist on a potential link between the reduction of apoC-III and CVD outcomes from randomized clinical trials of fibrates. Statins are reported to inhibit *APOC3* mRNA levels in liver cells [78] and a small reduction of apoC-III levels by statins has been reported in several relatively small studies [11, 79, 80].

The strategy to search for naturally occurring loss-of-function mutations that associate with decreased cardiovascular disease has proven successful for identifying novel therapeutic targets. For example, the translation of findings on proprotein convertase subtilisin/kexin type 9 (PCSK9) variants, from their identification in 2003 [81] to the currently ongoing phase 3 clinical outcome trials of PCSK9 inhibition with almost 40 000 persons included [82], has been performed in record time. Keys for this rapid translation from bench to clinic encompass the development of modern genetics and new techniques such as silencing RNA and novel human antibody technology [83]. ApoC-III follows the same modern pipeline for drug discovery, as ISIS Pharmaceuticals has developed an apoC-III ASO (volanesorsen) that inhibits the biosynthesis of apoC-III [46, 84]. ApoC-III ASO has been shown to robustly reduce both plasma apoC-III and triglyceride levels in multiple animal models and human volunteers in phase I studies [46]. Recent data from phase II studies confirmed that apoC-III ASO-mediated suppression of apoC-III leads to marked lowering (up to 90 %) of serum apoC-III and up to about an 80 % reduction of VLDL triglyceride levels in patients with familial chylomicron syndrome and with variable types of hypertriglyceridemia [46, 85••, 86••]. The data also demonstrated that the lowering of serum apoC-III levels and triglycerides by apoC-III ASO was dose-dependent and selective [46]. In addition, volanesorsen markedly reduced TRL remnants [85••]. These observations, together with the reduction of TRLs in the absence of LPL, support the existence of LPL-independent pathway(s) regulated by apoC-III for removal of triglycerides [86••]. Safety data from these short-term studies with apoC-III ASO therapy have not raised any major concerns; volanesorsen appears to have a good safety profile and has been well tolerated [85••, 86••]. Phase III trials with volanesorsen are ongoing (the APPROACH study and COMPASS) and will clarify the efficacy of the drug intervention in subjects with severe hypertriglyceridemia defined as triglyceride levels above 5.7 mmol/l (>500 mg/dl) [87].

### Gaps in Knowledge of ApoC-III

Important contributions to our understanding of apoC-III have been made by several research groups. However, there are still important gaps in our knowledge of apoC-III (see Table 1), the closing of which will require additional research.

**Table 1:** Gaps in our knowledge of apoC-III

1. Importance of apoC-III for VLDL secretion in humans
2. Role of apoC-III isoforms for function and pathophysiology
3. Factors regulating apoC-III expression: role of hormones and external factors
4. Role of apoC-III on HDL and HDL subspecies
5. ApoC-III as a potential biomarker of the residual risk of CVD
6. Role of apoC-III in intestinal lipid metabolism

## Conclusions

Population studies have demonstrated that plasma apoC-III is strongly associated with CVD risk. For many years, the atherogenicity of apoC-III was attributed to its LPL-mediated effects. However, recent evidence reveals that apoC-III has additional roles, for example in promoting VLDL formation and assembly and proinflammatory responses in endothelial cells and monocytes and impairing LPL-independent hepatic clearance of TRL remnants. Encouraging results from early clinical trials demonstrate that modulation of apoC-III per se is a novel and potent therapeutic approach to managing dyslipidemia and CVD risk.
